# S04-5 Development of the Sports Club for Health (SCforH) online learning tool

**DOI:** 10.1093/eurpub/ckac093.021

**Published:** 2022-08-29

**Authors:** Danijel Jurakic, Tena Matolic, Zeljko Pedisic

**Affiliations:** 1 University of Zagreb, Faculty of Kinesiology, Zagreb, Croatia; 2 Victoria University, Institute for Health and Sport, Melbourne, Australia

**Keywords:** Dissemination, Sport clubs, Sport associations, Exercise professionals

## Abstract

**Background:**

In 2019, the European Commission provided funding of nearly 400 thousand Euro for the third round of the Sports Club for Health (SCforH) project. The objective of this project is to increase participation in sport and HEPA in the European Union by encouraging sports clubs and associations to implement SCforH principles in their activities. To achieve the objective of the project, in this study we aimed to develop a SCforH online learning tool.

**Methods:**

Through a comprehensive literature search and consultations with the stakeholders in the area of sport, we created a list of examples of good practice in implementing SCforH initiatives and other related HEPA promotion practices in the sports context. The list was used to inform the development of the SCforH online learning tool. The SCforH online learning tool was developed through a consultation process including 15 experts in the area of sport and health.

**Results:**

The SCforH online learning tool includes: a participation consent form; a module for setting learning objectives, textual, pictorial, and video learning materials; interactive learning exercises; in-course quizzes and self-assessment modules; a link to the database of SCforH initiatives and other related practices in the European Union; links to other available online materials for those who want to know more; a module for the assessment of acquired knowledge; a participant feedback module; an optional survey including questions relevant for the evaluation of SCforH activities.

**Conclusions:**

The SCforH online learning tool will enable the realisation of other aims of the current SCforH project, in particular, raising the awareness of the SCforH guidelines among: European sports clubs; sport associations; HEPA promoters; policymakers; and students of physical education, sport science, and public health.

